# Description of the Early Development of *Hoplias intermedius* (Günther, 1864) (Characiformes: Erythrinidae)

**DOI:** 10.1002/jmor.70151

**Published:** 2026-07-23

**Authors:** Mateus Babichi Veiga de Souza, Renan Souza Volpato, Andréa Bialetzki

**Affiliations:** ^1^ Laboratório de Ictioplâncton, Nupélia (Núcleo de Pesquisas em Limnologia, Ictiologia e Aquicultura), Centro de Ciências Biológicas (CCB) Universidade Estadual de Maringá (UEM) Maringá Brazil; ^2^ Departamento de Biologia Programa de Pós‐graduação em Ecologia de Ambientes Aquáticos Continentais (PEA), (DBI‐CCB‐UEM) Maringá Brazil

**Keywords:** external morphology, ichthyoplankton, ontogeny, stages of development, taxonomy

## Abstract

In this study, we provide a detailed description of the eggs, larvae, and juveniles of *Hoplias intermedius*, focusing on their morphological, meristic, and morphometric characteristics. Specimens were obtained through induced spawning using the hypophysation technique. The eggs (*n* = 82) were large and spherical, with a yellowish yolk, transparent chorion, and a small perivitelline space. During development, 135 larvae and 16 juveniles were examined, with standard lengths ranging from 5.48 to 39.00 mm. Larvae exhibited a terminal mouth, simple nostrils, spherical eyes, a large yolk sac, occupying approximately one‐third of the body, and an intestine extending beyond the mid‐body region. Pigmentation was initially sparse but became more intense during the preflexion stage. Juveniles already exhibited body morphology similar to that of adults. Fin rays counts varied as follows: pectoral (9–10), pelvic (7–9), dorsal (12–14), anal (10–13), and caudal (18–20). The total number of myomeres ranged from 36 to 44, with a modal distribution of 28 preanal and 16 postanal myomeres. Morphometric analysis revealed variation in body height (from elongated to moderate), head length (from small to large), and eye diameter (from moderate to large). Throughout development, increases were observed in snout length, head height, and distances from the snout to the pectoral, pelvic, dorsal, and anal fins. Growth analyzes indicated that most morphometric variables changed progressively during ontogeny, although some exhibited shifts in growth trajectories (breakpoints), likely associated with major developmental transitions such as yolk absorption and fin differentiation. These findings expand knowledge on Neotropical fish development. Specifically, the convergence of the lower margins of the dentary bones toward the mandibular symphysis provides a reliable diagnostic character to distinguish *Hoplias intermedius* from its congeners. This study offers essential morphological and meristic criteria for taxonomic identification, with direct implications for aquaculture and biodiversity conservation.

## Graphical abstract

This study provides the first description of the early development of *Hoplias intermedius* (Characiformes: Erythrinidae), from the embryonic to the juvenile stage. The morphological, meristic, and morphometric characterization contributes to understanding the ontogeny and developmental transitions of Neotropical fishes, supporting taxonomy, conservation, and aquaculture management.

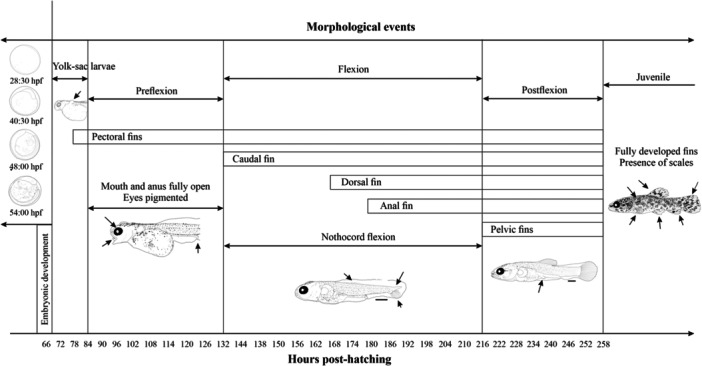

## 1 Introduction

Fishes represent the most diverse group of vertebrates on the planet, occupying a wide range of aquatic environments and playing fundamental ecological, economic, and social roles worldwide (McKenzie et al. 2024; Mello et al. 2025). Freshwater ecosystems, in particular, harbor extraordinary fish diversity, especially in the Neotropical region (Albert et al. 2020; Tonella et al. 2023). More than 6000 freshwater fish species have been formally described, although estimates suggest that between 8000 and 9000 species may occur in South America alone (Reis et al. 2016; Albert et al. 2020). This remarkable diversity is expressed through a wide array of morphologies, behaviors, life history strategies, evolutionary histories, and ecological functions (Winemiller 1989; Blanck et al. 2007; Vitule et al. 2017; Pelicice et al. 2021).

Among freshwater fishes, the order Characiformes stands out because of its wide distribution, high abundance, and remarkable morphological diversity, comprising one of the largest and most ecologically diverse groups of Neotropical fishes (Toledo‐Piza et al. 2024). Despite the significant number of Characiformes species with described early ontogeny (Reynalte‐Tataje et al. 2020), some families remain poorly studied with respect to developmental morphology, particularly Erythrinidae.

This family comprising 18 valid species and is characterized by a cylindrical body with cryptic coloration that aids camouflage, a broad mouth bearing caniniform teeth of variable sizes, a short anal fin, an adipose fin, and a rounded caudal fin (Guimarães et al. 2021; Gimênes Junior and Rech 2022; Froese and Pauly 2025). Erythrinids are exclusively distributed in South America and occur across a wide variety of freshwater environments (Gimênes Junior and Rech 2022). Commonly referred to as trahiras or giant trahiras, these species exhibit distinctive live‐history traits, such as nest building along riverbanks, parental care, and batch spawning (Nakatani et al. 2001; Diniz et al. 2023). Moreover, in many regions, they are economically and culturally important to artisanal fisheries and the ornamental fish trade (Oyakawa and Mattox 2009; Gimênes Junior and Rech 2022). The family includes three genera—*Erythrinus*, *Hoplerythrinus*, and *Hoplias*—with *Hoplias* being the most species‐rich and taxonomically complex genus (Mattox et al. 2014; Gimênes Junior and Rech 2022; Froese and Pauly 2025).

In Brazil, eight species of the genus *Hoplias* are currently recognized, among which *Hoplias intermedius* (giant trahira) stands out because of its ecological relevance and aquaculture potential (Faria et al. 2019). This species is valued for its adaptability to captivity, rapid growth, high‐quality meat, and popularity among sport fishers (Luz et al. 2000; Oyakawa and Mattox 2009; Ramos et al. 2018). Endemic to the São Francisco River basin, *H. intermedius* was formerly misidentified as *Hoplias lacerdae* and occurs sympatrically with its cogener *Hoplias* gr. *malabaricus* (Oyakawa and Mattox 2009; de M. C. Sassi et al. 2021). It inhabits lotic environments and can attain larger sizes than other congeners, such as *Hoplias malabaricus*, reaching up to one meter in length and 20 kg in weight (Castagnolli and Cyryno 1986; Diniz et al. 2023).

Adult *H. intermedius* can be distinguished from closely related species by specific meristic and morphometric traits, including the number of lateral line scales (42–46 in *H. intermedius* vs. 38–43 in *H. brasiliensis* and 34–39 in *H. curupira*), the number of pores in the laterosensory system on the ventral dentary surface (4–6 vs. 5 in H. australis and 6–8 in *H. lacerdae*), and the angular profile of the anterior of the head (vs. rounded in *H. australis*) (Oyakawa and Mattox 2009; Froese and Pauly 2025). However, many of these diagnostic characters are not fully developed or visible during the early life stages, making the identification of eggs, larvae, and juveniles considerably more challenging.

Descriptions of early developmental stages, traditionally associated with taxonomic identification, have increasingly incorporated biological approaches aimed at investigating growth patterns and morphological changes during ontogeny (Oliveira et al. 2024; Dos Santos et al. 2025). Fish development may occur through discrete phases marked by abrupt functional and morphological transitions, a process known as saltatory ontogeny (Balon 1984). Within this framework, analyzes of body proportions during early development help determine whether growth follows isometric or allometric patterns and allow the identification of developmental shifts associated with ecological and functional changes (Balon 1990; Kováč et al. 1999; Bialetzki et al. 2008; Cajado et al. 2023; Souza et al. 2023; Silva et al. 2024).

The precise identification of fish durint their early developmental stages is a critical step in ecological and conservation research. It also provides valuable insights for aquaculture and helps fill knowledge gaps regarding the early life history of fish, particularly in the Neotropical region (Reynalte‐Tataje et al. 2020; Souza et al. 2023; Oliveira et al. 2024). Despite the ecological and economic relevance of *H*. *intermedius*, no study has yet described the morphology of its eggs, larvae, and juveniles. Therefore, this study aims to provide a detailed description of the early development of *Hoplias intermedius*, focusing on external morphology, pigmentation patterns, and morphometric traits. Additionally, we analyze body proportions and growth patterns across developmental stages testing the hypothesis of differential development during the species' early ontogeny to better understand the ontogeny of the specie and contribute to comparative studies on the early development of Neotropical fishes.

## 2 Materials and Methods

### 2.1 Collection and Preparation of Biological Material

Specimens of *H. intermedius* were obtained through induced spawning using the hypophysation technique. The procedure was carried out at the Furnas Fish Farming Station, located in the municipality of São José da Barra, Minas Gerais, Brazil (20°40'36.6“S, 46°20'01.4“W), using sexually mature broadstook from the São Francisco River basin. These adult individuals were collected from local environments and subjected to induced reproduction in January 1998. Adult specimens were identified by taxonomists associated with the fish farming station.

To analyze and characterize the different early development periods and stages, eggs were collected immediately after hydration and subsequently at 2‐h intervals until hatching. Larvae were sampled from hatching until complete yolk‐sac absorption, at intervals ranging from 2 to 6 h, after collected every 12 h until the juvenile period, when sampling was performed daily. The sampling periodicity followed Nakatani et al. (2001). All the samples were fixed in 4% formalin solution buffered with calcium carbonate. Following collection, all specimens were transported to the Ichthyoplankton Laboratory at the Núcleo de Pesquisa em Ictiologia Limnologia e Aquicultura (Nupélia) of the Universidade Estadual de Maringá (UEM), where the material was incorporated into the Ichthyoplankton Collection and remained preserved in the same solution under regular monitoring of fixation conditions.

### 2.2 Analysis of Biological Material

For the analysis of early ontogeny, individuals were classified according to their developmental stage: embryonic (initial cleavage, early embryo, free‐tail embryo, and late embryo), larval (yolk‐sac, preflexion, flexion, and postflexion), and juvenile. This classifiction followed Ahlstrom and Moser (1976), as modified by Nakatani et al. (2001). Each developmental period and stage were described based on the degree of morphological deifferentiation and the occurrence of key developmental events. The most representative individuals were photographed and illustrated.

Representative specimens from all developmental periods and stages were deposited in the Coleção Ictiológica do Nupélia‐UEM, Paraná, Brazil (voucher number: NUP023247).

**Figure 1:**
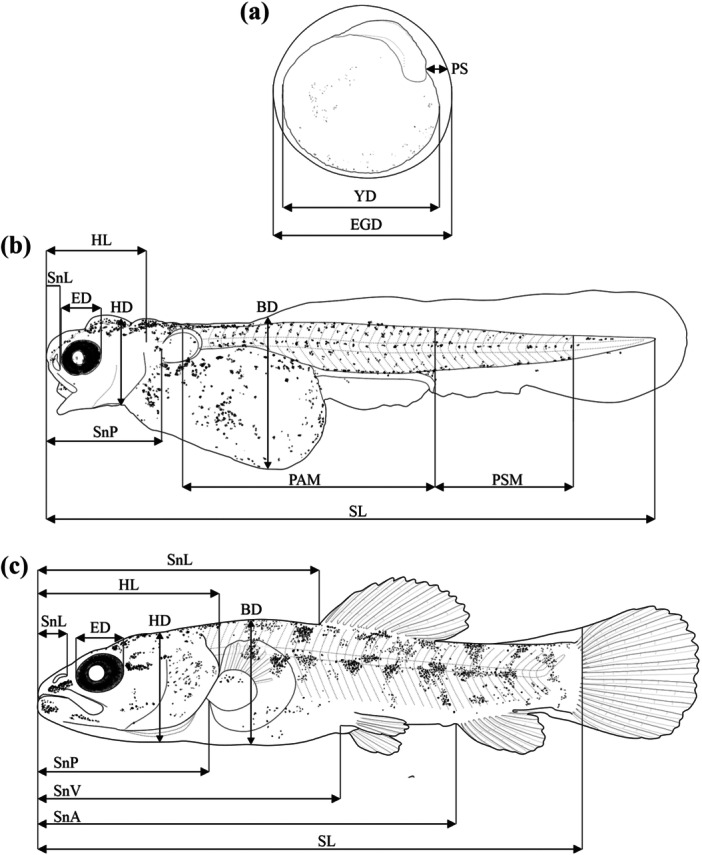
Morphometric measurements obtained in the (A) egg, (B) preflexion larvae, and (C) postflexion larva of *Hoplias intermedius*. BD, body height; ED, eye diameter; EGD, egg diameter; HD, head height; HL, head length; PS, perivitelline space; SL, standard length; SnA, snout‐anal fin distance; SnD, snout‐dorsal fin distance; SnL, snout length; SnP, snout‐pectoral fin distance; SnV, snout‐pelvic fin distance; YD, yolk sac diameter.

For the morphometric characterization of early development, measurements were obtained using a stereomicroscope equipped with OPHTD software (Opticam Microscopy Technology). The following variables, expressed in millimeters with a precision of 0.01 mm, were recorded: egg diameter (EGD), yolk sac diameter (YD), and perivitelline space (PS), the latter expressed as a proportion of the total egg volume (Figure 1a) (Nakatani et al. 2001).

Following hatching, additional morphometric measurements were taken along the longitudinal axis of the body, in accordance with Ahlstrom and Moser (1976). These included: standard length (SL), snout length (SnL), eye diameter (ED), head height (HD), head length (HL), body height (BD), and distances snout‐pectoral fin (SnP), snout‐pelvic fin (SnV), snout‐dorsal fin (SnD), and snout‐anal fin (SnA) (Figure 1c).

In yolk‐sac and preflexion larvae, in which the caudal fin is not yet formed, body length was measured as notochord length, defined as the distance from the tip of the snout to the end of the notochord (Figure 1b). In flexion and postflexion larvae, as well as juveniles, standard length was measured from the tip of the snout to the hypural plate (Figure 1c) (Ahlstrom and Moser 1976; Nakatani et al. 2001; Oliveira et al. 2024; Silva‐Cajado et al. 2025).

For the meristic characterization, counts were made, whenever possible, of the total, preanal, and postanal myomeres, as well as the number of fin rays in the pectoral (P), pelvic (V), dorsal (D), anal (A) and caudal (C) fins (Figure 1b). All morphometric and meristic variables anlyzed in this study are summarized in Figure 1.

### 2.3 Data Analysis

To analyze morphometric relationships as proportions, head height (HD), snout length (SnL), and eye diameter (ED) were expressed relative to head length (HL), whereas head length (HL), body height (BD), HD, and the distances from the snout to the pectoral (SnP), pelvic (SnV), dorsal (SnD), and anal (SnA) fins were expressed relative to standard length (SL). The classification of morphometric relationships associated with body depth (BD/SL), head length (HL/SL), and eye diameter (ED/HL) followed the criteria proposed by Leis and Trnski (1989).

To examine ontogenetic variation during development, morphometric variables (response variables) were plotted against standard length and head lengths, which were as explanatory variables. These relationships were analyzed using different regression models following Kováč et al. (1999). Initially, the hypothesis that body proportion development follows continuous isometry was tested using simple linear regression. In addition to the continuous isometry hypothesis, alternative hypotheses were evaluated, including gradual allometric development (quadratic regression) and discontinuous isometry (piecewise linear regression characterized by breakpoints indicating shifts in growth rates). The best model for each morphometric variable relative to body and head size was selected using F‐tests (Sokal and Rohlf 1981). A significance level of *p* < 0.05 was adopted for all analyzes. Statistical analyzes were performed in R software, version 4.1.1 using the Segmented package (Muggeo 2008).

## 3 Results

### 3.1 Embryonic Development

**Figure 2:**
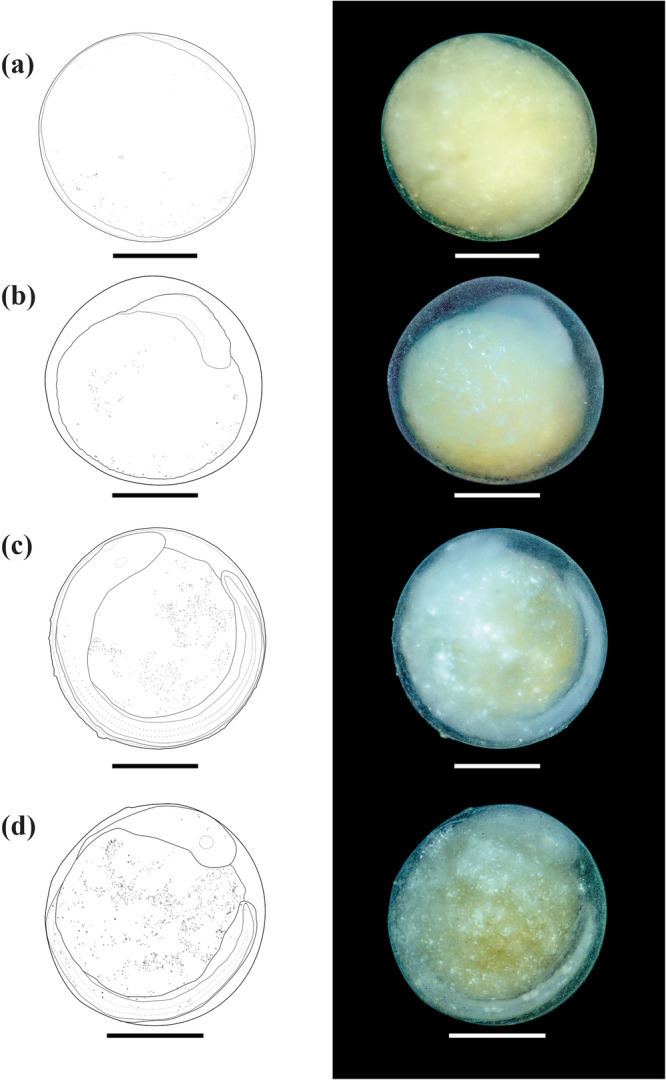
Embryonic development of *Hoplias intermedius*. (a) Initial cleavage (28:30 hpf), (b) Early embryo (40:30 hpf), (c) Free‐tail (48:00 hpf), and (d) Late embryo (54:00 hpf). Scale bar = 1 mm; hpf = hours post‐fertilization.

The embryonic period lasted approximately 66 h at an average water temperature of 28°C. During this period, a total of 82 eggs were analyzed and classified into the following developmental stages: initial cleavage (*n* = 20), early embryo (*n* = 23), free‐tail (*n* = 31), and late embryo (*n* = 8). The morphometric and meristic data are presented in Table 1. A detailed morphological description of each developmental stage is provided and illustrated in Figure 2.

**Table 1:** Minimum (Min), maximum (Max), mean (X), and standard deviation (SD) values (mm) for morphometric variables and meristic counts obtained from eggs of *Hoplias intermedius*. CI, initial cleavage; CL, free‐tail; EF, late embryo; EGD, egg diameter; EI, early embryo; PS, perivitelline space; YD, yolk diameter.

Variables (mm)	CI (*n* = 20)		EI (*n* = 23)		CL (*n* = 31)		EF (*n* = 8)	
	Min–max	X–SD	Min–Max	X–SD	Min–Max	X–SD	Min–Max	X–SD
**EGD**	2.11–2.49	2.40–0.13	2.22–2.61	2.44–0.10	2.20–2.71	2.47–0.12	2.29–2.58	2.45–0.08
**YD**	1.82–2.57	2.20–0.22	1.84–2.36	2.13–0.15	1.84–2.49	2.11–0.17	1.79–2.46	2.15– 0.20
**PS**	0.13–0.49	0.29–0.10	0.11–0.41	0.24–0.09	0.10–0.59	0.23–0.11	0.17–0.37	0.25–0.07

The eggs were large and spherical, with a yellowish yolk, transparent chorion and small perivitelline space. Throughout the embryonic period, no significant changes in size were observed (EGD = 2.11–2.71 mm; YD = 1.79–2.57 mm; and PS = 0.10 0.59 mm) (Table 1).

During the initial cleavage stage (21 h 30 min post‐fertilization), the yolk remains localized at the vegetal pole, whereas cleavage occured at the animal pole. The cleavage pattern was discoidal meroblastic (Figure 2a). The early embryo stage (36h30min post‐fertilization) was characterized by cephalocaudal differentiation and the establishment of bilateral symmetry, withthe embryo positioned dorsally over the yolk (Figure 2b).

At the free‐tail stage (44 h 30 min post‐fertilization), the tail became detached from the yolk, defining the future body axis of the larva (Figure 2c). During the the late embryo stage (50h30min post‐fertilization), the embryos were fully developed and resemble pre‐hatching larvae. The eyes were visible (Figure 2d) but lacked pigmentation. This period concluded with hatching, when the embryo broke through the chorion about 66 h post‐fertilization.

### 3.2 Larval Development

**Figure 3:**
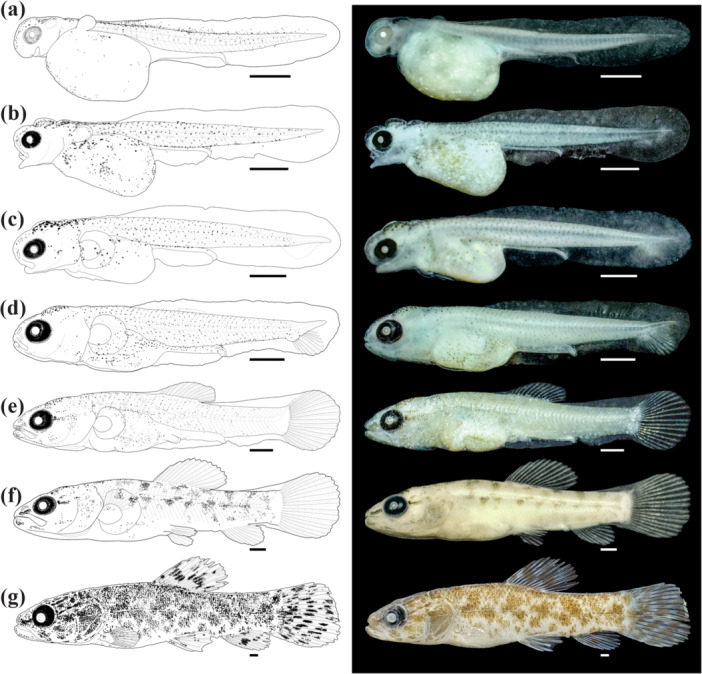
Early development of *Hoplias intermedius*, (a) yolk‐sac larvae (81:30 hph), (b) preflexion (128:00 hph), (c) early flexion (144:00 hph), (d) late flexion (186:30 hph), (e) early postflexion (222:00 hph), (f) late postflexion (254:00 hph), and (g) juvenile (> 258 hph). Scale bar = 1 mm; hph, hours post‐hatching.

**Figure 4:**
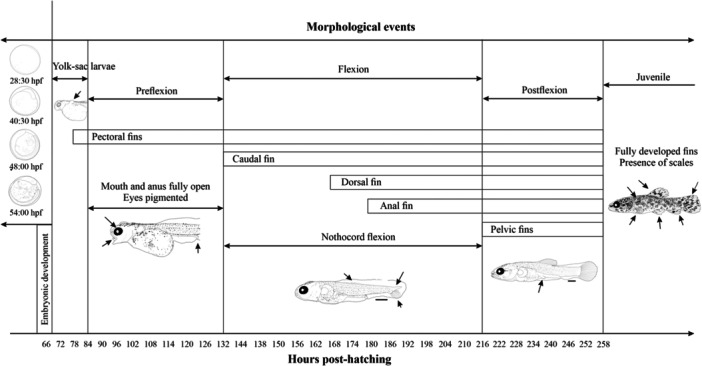
Summary of morphological events observed during the early development of *Hoplias intermedius*. Embryonic development is indicated in hours post‐fertilization (hpf), whereas larval stages are incated in hours post‐hatching. Hatching occurred at approximately 66 hpf.

A total of 135 larvae were analyzed during larval period, with standard lenght ranging from 5.48 to 28.55 mm. Of these, 24 were classified as yolk‐sac stage, 18 as preflexion stage, 37 as flexion stage, and 56 as postflexion stage. Morphometric and meristic data are presented in Table 2. The main morphological changes observed during the early development, as well as the description of each developmental stage of *Hoplias intermedius*, are summarized in Figures 3 and 4.

**Table 2:** Minimum (Min), maximum (Max), mean (X), mode (Mo), and standard deviation (SD) (mm) for morphometric variables, body relations (%) and meristic counts in larvae and juveniles of *Hoplias intermedius*. BD, body height; ED, eye diameter; FL, flexion stage; FP, postflexion stage; HD, head height; HL, head length; J, juveniles; NV, not visible; PF, preflexion stage; SL, standard length; SnA, snout‐anal fin distance; SnD, snout‐dorsal fin distance; SnL, snout length; SnP, snout‐pectoral fin distance; SnV, snout‐pelvic fin distance; YS, yolk‐sac larvae; –, fin absent.

Stages										
Variables (mm)	Larval period								Juvenile period	
	YS (*n* = 24)		PF (*n* = 18)		FL (*n* = 37)		FP (*n* = 56)		J (*n* = 16)	
	Min–Max	X–SD	Min–Max	X–SD	Min–Max	X–SD	Min–Max	X–SD	Min–Max	X–SD
SL	5.84–7.72	6.85–0.57	6.97–8.44	7.90–0.35	8.13–10.69	9.04–0.66	10.70–28.55	17.21–5.70	26.60–39.00	32.09–3.46
SnL	0.16–0.26	0.22– 0.03	0.18–0.29	0.24–0.03	0.23–0.58	0.35–0.08	0.56–2.70	1.22–0.63	2.10–3.55	2.99–0.40
ED	0.17–0.54	0.36–0.12	0.41–0.64	0.56–0.06	0.58–0.89	0.73–0.07	0.90–3.10	1.57–0.61	2.55–3.55	2.99–0.25
HL	0.39–1.18	0.76– 0.24	0.87–1.52	1.26–0.19	1.31–2.68	1.80–0.38	2.68–8.85	4.97–1.86	7.95–10.75	9.24–0.72
HD	0.35–1.20	0.77–0.27	0.86–1.42	1.23–0.14	1.36–1.99	1.60–0.16	1.99–6.55	3.39–1.15	5.20–7.10	6.04–0.54
BD	1.70–1.93	1.79–0.06	1.52–2.00	1.78–0.12	1.56–2.27	1.80–0.19	2.27–7.50	3.95–1.49	6.40–9.25	7.42–0.80
SnP	1.39–1.70	1.52–0.14	1.30–2.03	1.69–0.20	1.68–3.01	2.15–0.37	3.01–10.00	5.52–2.11	9.35–12.95	10.72–1.11
SnV	—	—	—	—	—	—	5.91–17.50	9.80–3.33	16.35–21.50	18.14–1.76
SnD	—	—	—	—	4.21–5.84	4.77–0.37	5.88–15.00	8.92–2.79	14.60–19.00	16.55–1.46
SnA	—	—	—	—	6.35–8.01	6.85–0.49	8.03–22.40	13.44–4.54	20.75–29.90	24.89–2.85
Relations (%)										
SnL/HL	19.44–45.45	30.79–9.08	13.53–28.74	19.63–4.23	17.16–25.66	19.74–1.99	19.21–34.38	23.56 –3.96	25.86–37.85	32.38–3.33
ED/HL	40.48–56.94	47.67–4.25	37.76–51.00	44.89–3.86	32.09–49.64	41.76–4.96	26.51–40.28	31.66 –3.28	28.81–37.57	32.43–2.35
HD/HL	81.03–126.98	100.83–11.97	84.46–111.88	98.10–7.49	72.53–107.52	91.37–10.48	60.47–83.33	69.49 –6.10	54.50–77.99	65.66–6.81
HL/SL	6.68–15.28	10.91–2.76	12.48–18.34	15.88–1.87	15.94–25.07	19.68–2.72	24.00–33.05	28.49–2.44	24.23–32.71	28.92–1.97
BD/SL	23.13–31.68	26.26–2.42	18.38–26.46	22.63–2.03	17.35–24.48	19.89–1.36	19.92–26.50	22.63–1.57	20.36–27.07	23.18–1.52
SnP/SL	18.29–22.02	20.02–1.69	16.95–24.61	21.17–1.98	19.93–28.65	23.63–2.28	28.06–35.73	31.59–2.10	30.65–36.84	33.46–1.75
SnV/SL	—	—	—	—	—	—	51.77–61.30	56.79–1.86	52.63–62.97	56.65–2.46
SnD/SL	—	—	—	—	48.56–55.29	52.18–1.99	49.00–55.21	52.14–1.78	48.46–54.94	51.71–2.03
SnA/SL	—	—	—	—	71.08–75.64	72.91–1.37	74.25–82.61	77.91–2.08	73.99–81.81	77.52–1.88

Rays										
	Min–Max	Mo–SD	Min–Max	Mo–SD	Min–Max	Mo–SD	Min–Max	Mo–SD	Min–Max	Mo–SD
Pectoral	NV	NV	NV	NV	NV	NV	5.00–9.00	8.00–1.76	9.00–13.00	11.00–0.90
Ventral	—	—	—	—	—	—	6.00–8.00	8.00–0.77	7.00–9.00	8.00–0.35
Dorsal	—	—	—	—	—	—	13.00–14.00	14.00–0.63	12.00–14.00	13.00–0.61
Anal	—	—	—	—	—	—	11.00–13.00	10.00–1.34	10.00–13.00	11.00–0.87
Caudal	—	—	—	—	—	—	17.00–19.00	18.00–1.87	18.00–20.00	18.00–0.69
Myomeres										
Pre‐anal	21.00–25.00	24.00–1.14	23.00–28.00	25.00–1.51	24.00–29.00	27.00–1.12	24.00–29.00	27.00–2.33	NV	NV
Post‐anal	14.00–16.00	16.00–0.76	15.00–19.00	16.00–1.81	15.00–19.00	16.00–1.48	16.00–19.00	18.00–1.18	NV	NV
Total	36.00–41.00	39.00–1.05	37.00–44.00	43.00–2.23	39.00–44.00	41.00–1.63	39.00–44.00	41.00–2.63	NV	NV

#### 3.2.1 Yolk‐sac Larvae (Table 2; Figure 3a)

This stage extended from hatching until the onset of eye pigmentation and the opening of the mouth and anus. Larvae exhibited standard lengths ranging from 5.84 to 7.72 mm (6.85 mm SL ± 0.57). The yolk sac was relatively large, occupying approximately one‐third of the body lenght. The mouth and anus were closed. The eyes were spherical, and the notochord was straight straight and visible because of body transparency. An embryonic finfold extended from the postcephalic region to the posterior edge of the yolk sac. Some pigmentation appeared on the dorsal region of the head and along the body and finfold in specimens measuring 6.85 mm onward. Pectoral fin buds were evident in individuals measuring approximately 7.43 mm SL. The myomeres total number rangeded from 36 to 41, with a modal count of 24 preanal and 16 postanal myomeres.

#### 3.2.2 Preflexion Stage (Table 2; Figure 3b)

Standard length ranged from 6.97 to 8.44 mm (7.90 mm SL ± 0.35). At this stage, the mouth and anus were fully open, and the eyes were pigmented. The yolk sac was partially absorbed; the intestine was long, with the anus positioned posterior to the mid‐body region. The notochord remained straight and visible because of body transparency. The embryonic finfold was still present. Pigmentation became more intense on the dorsal region of the head and dorsolaterally. Pigments also persisted in the finfold, with a predominance and intensification of dendritic chromatophores. The pectoral fin bud was evident. The total number of myomeres ranged from 37 to 44, with a modal count of 25 preanal and 16 postanal myomeres.

#### 3.2.3 Flexion Stage (Table 2; Figure 3c–d)

The standard length of larvae at this stage ranged from 8.13 to 10.69 mm (9.04 mm SL ± 0.66). Traces of the yolk sac could still be observed in individuals measuring approximately 8.59 mm SL, but it became completely absorbed by the end of this stage. The mouth was terminal, and pigmentation increased in intensity compared with the preflexion stage, especially along the lateral region of the body and the dorsal region of the head. The notochord was flexed and remained visible because of body transparency. In contrast to the previous stage, the first caudal‐fin rays became apparent at 9.04 mm SL, followed by the initial development of dorsal‐ and anal‐fin rays at 9.35 mm SL and 10.41 mm SL, respectively. The total number of myomeres ranged from 39 to 44, with a modal count of 27 preanal and 16 postanal myomeres.

#### 3.2.4 Postflexion Stage (Table 2; Figure 3e–f)

Larvae at this stage exhibited standard lengths ranging from 10.70 to 28.55 mm (17.21 mm SL ± 5.70). The mouth was terminal, with canine teeth visible on the dentary and premaxilla and the nostrils were simple. The finfold was nearly completely absorbed; however, remnants could still be observed in the dorsal region between the dorsal and caudal fins, as well as in the ventral region between the end of the abdominal area and the caudal fin, in individuals up to approximately 14.30 mm SL. At this stage, the increased pigmentation and epidermal development obscured the myomeres and prevented visualization of the the notochord and swim bladder through body transparency. Pigmentation was concentrated in the head region and distributed along the dorsal and lateral surfaceas of the body, as well as between the fin rays of the dorsal and caudal fins. The pelvic fin bud emerged during this stage, with fin rays becoming visible in individuals measuring approximatelly14.00 mm SL. Pectoral‐fin rays were already visible, and by the end of this stage all fins exhibited developed rays. The number of fin rays was as follows: pectoral = 5–9, pelvic = 6–8, dorsal = 13–14, anal = 11–13, and caudal = 17–19. The total number of myomeres ranged from 39 to 44, with a modal count of 27 preanal and 18 postanal myomeres.

### 3.3 Juvenile Development

A total of 16 individuals were analyzed during this period, with a standard lengths ranging from 26.60 to 39.00 mm (mean ± SD = 32.09 ± 3.46) (Table 2; Figure 3g). Juveniles exhibited body morphology similar to that of adults, including fully developed fins, the presence of scales, and a terminal mouth bearing visible canine teeth on both the dentary and premaxilla. The body and head displayed extensive and irregular, and the fins and eyes were fully pigmented. The embryonic fin was completely reabsorbed, and myomeres were no longer visible because of the loss of body transparency. The number of fin rays observed in this period was as follows: pectoral = 9–10, pelvic = 7–9, dorsal = 12–14, and anal = 10–13, and caudal 18–20.

### 3.4 Morphometric Relations

Morphometric variables associated with standard length exhibited proportional changes throughout ontogeny. Body height varied from elongated to moderate (BD 17.35%–31.68% SL), whereas head length ranged from small to large (HL 6.68%–33.05% SL). Distances from the snout to the pectoral, pelvic, dorsal, and anal fins increased progressively throughout development (Table 2). Among the variables related to head length, eye diameter varied from large to moderate (ED 26.51%–56.54% HL), whereas snout length and head height exhibited positive growth trends (Table 2).

### 3.5 Growth Analyzes

**Figure 5:**
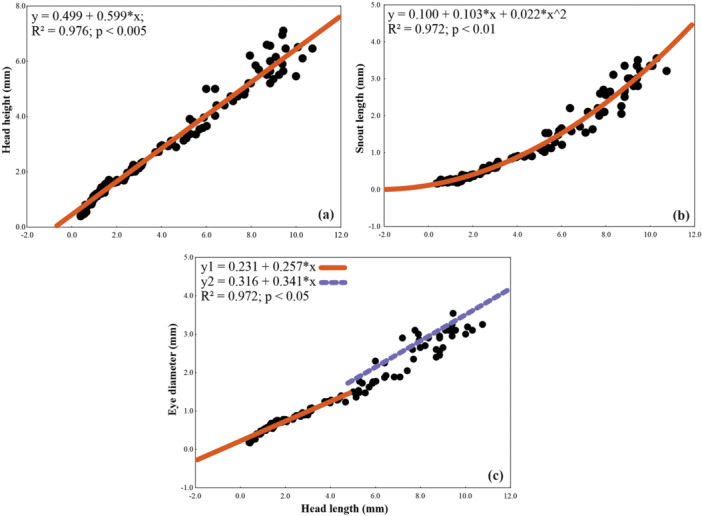
Morphometric relationships (mm) between head length and: a. head height, b. eye diameter, and c. snout length during the early development of *Hoplias intermedius*.

The growth pattern of head depth (Figure 5a) was best described by a linear regression, whereas snout length (Figure 5b) followed a quadratic model and eye diameter (Figure 5c) was best explained by a piecewise linear regression model, all in relation to head length (Table 3; Figure 5).

**Table 3:** Linear, quadratic, and piecewise regression statistics for morphometric variables in relation to head length and standard length of larvae and juveniles of *Hoplias intermedius*. BM, best model; BP, breakpoint; L, linear regression; N, number of individuals analyzed; Q, quadratic regression; R², coefficient of determination; S, piecewise regression.

Relation	R^2^L	R^2^S	R^2^Q	F Q/L	*p*	F S/Q	*p*	F S/L	*p*	BM	BP	*N*
SnL/HL	0.95	0.97	0.97	122.93	0.00	1.76	0.19	62.66	0.00	Q	4.85	151
ED/HL	0.97	0.97	0.97	9.66	0.00	7.68	0.01	8.89	0.00	S	4.82	151
HD/HL	0.98	0.98	0.98	0.51	0.48	11.42	0.57	5.98	0.00	L	9.40	151
HL/SL	0.97	0.99	0.99	637.80	0.00	−83.34	0.00	94.26	0.00	Q	3.61	151
BD/SL	0.98	0.98	0.98	0.00	0.97	24.47	0.00	12.23	0.00	S	3.24	151
SnP/SL	0.99	0.99	0.99	58.65	0.00	−1.21	0.27	28.22	0.00	S	4.58	134
SnV/SL	0.99	0.99	0.99	6.52	0.01	1.77	0.19	4.18	0.04	S	12.03	71
SnD/SL	0.99	0.99	0.99	3.09	0.08	2.12	0.15	2.62	0.11	L	9.12	101
SnA/SL	1.00	1.00	1.00	8.81	0.00	6.02	0.02	7.66	0.01	S	14.22	93

**Figure 6:**
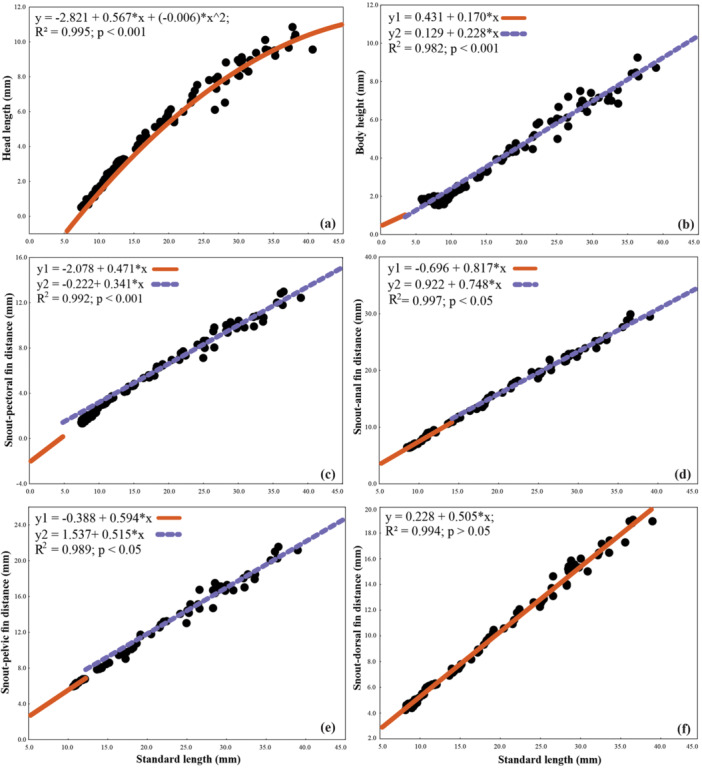
Morphometric relationships (mm) between standard length and: a. head length, b. body height, c. snout‐pectoral fin distance, d. snout‐anal fin distance, e. snout‐pelvic fin distance, and f. snout‐dorsal fin distance during the early development of *Hoplias intermedius*.

In relation to standard length (Table 3; Figure 6), head length (Figure 6a) was best represented by a quadratic model, whereas snout‐dorsal fin distance (Figure 6f) was best fitted by a linear regression, and body depthand the distances form the snout to the pectoral, pelvic, and anal fins (Figure 6b–e) were best described by piecewise linear regression. These results indicate that growth initially followed a consistent pattern; however, once larvae reached a certain size (i.e., the breakpoint), marked shifts in growth trajectory were observed.

## 4 Discussion

In this study, we described the early development of *H. intermedius*, a native species of the Erythrinidae from the São Francisco River basin. As observe in other congeners, *H. intermedius* exhibits external fertilization, adhesive eggs, and parental care (Nakatani et al. 2001; Diniz et al. 2023). Early development is altricial (Balon 2006), with larvae hatching in a poorly developed, and small and transparent. During ontogeny, individuals progressively acquire a more robust morphology and gradually develop adult‐like features, reflecting the species ontogenetic transition and characteristic growth pattern.

The embryonic development of *H. intermedius* lasted 66 h at 28°C, which is relatively longer than that reported for *Hoplias* aff. *malabaricus*, whose embryos hatch in approximately 44 h at 24–26.5°C (Nakatani et al. 2001). In species whth adhesive eggs, embryogenesis generally lasts 41–46 h at 23°C–24°C (Rizzo and Godinho 2003). Although higher temperatures tend to accelerate this process (Reynalte‐Tataje et al. 2011), the longer developmental period observed in *H. intermedius* appears to be associated with egg size. According to Rizzo and Godinho (2003), fish eggs are considered large when their diameter ranges from 1.0 to 3.0 mm, and the eggs of *H. intermedius* measured between 2.11 and 2.58 mm. The relatively large yolk volume likely requires a longer period for embryonic differentiation, helping to explain the prolonged development observed even at higher temperatures.

The eggs were spherical, with a transparent chorion and yellowish yolk, characteristics consistent with those reported for other species of *Hoplias* species (Nakatani et al. 2001). The absence of a marked reduction in egg size throughout embryogenesis suggests limited yolk absorption before hatching, reflecting an energy allocation strategy that favors the use of yolk reserves in the larval stage (Godinho 2007). This interpretation is further corroborated by the similarity between late embryos and newly hatched larvae, consistent with an altricial developmental pattern in which larvae remain highly dependent on yolk reserves before te onset of exogenous feeding.

The presence of a large yolk sac may enhance early developmental performance by extending the endogenous feeding period and supporting morphofunctional development during the initial stages of life (Blaxter 1988; Deslauriers et al. 2018). In species such as *Hoplias intermedius*, early survival may also be influenced by parental care, which is known to occur in the genus (Nakatani et al. 2001; Gimênes Junior and Rech 2022). As development progress, pigmentation of the eyes and the opening of the mouth and anus during the preflexion stage indicate the onset of exogenous feeding, even while yolk reserves are still present. This transitional phase, characterized by mixed feeding, is crucial for larval survival (Kamler 1992). Ontogenetically, trahiras are planktivorous during the larval stage, insectivorous during the juvenile stage, and piscivorous in adulthood (Bialetzki et al. 2008; Gimênes Junior and Rech 2022). Morphological changes in the mouth, such as the development of the premaxillary, maxillary, and dentary bones, likely reflect this tropic shift (Bialetzki et al. 2008). Furthemore, the terminal position of the mouth suggests that feeding occurs predominantly within the water column (Bemvenuti and Fischer 2010).

Efficient feeding also depends on locomotor capacity (Rice and Hale 2010). Fin development follows a sequential pattern: the pectoral fins appear first, providing basic stability, followed by differentiation of the caudal fin, which supports propulsion, and later by the dorsal, anal, and pelvic fins, which contribute to stability and maneuverability (Webb and Weihs 1986; Bemvenuti and Fischer 2010). Although the pectoral fins are the first to appear, they are the last to complete fin‐ray formation because their function is more closely related to fine locomotor control than to primary propulsive, a pattern typically found in Characiformes (Webb and Weihs 1986; Nakatani et al. 2001; Galuch et al. 2003; Souza et al. 2023; Ito et al. 2025). This sequence reflects the functional demands imposed by the larval hydrodynamic environment. In early stages, propulsion relies primarily on body undulations, whereas the progressive differentiation of the fins improves swimming stability, maneuverability, and control (Webb and Weihs 1986; Santos et al. 2020). Even in species associated with calmer marginal habitats, such as *Hoplias intermedius*, these changes enhance the larvae's ability to maintain position in the water column, perform short displacements, and execute rapid strike movements during feeding and predator avoidance (Webb and Weihs 1986; Maciel et al. 2009; Santos et al. 2020).

Pigmentation patterns are valuable for species identification because each species exhibits a distinct pattern, with melanophore size and distribution being genetically determined (Nakatani et al. 2001; Andrade et al. 2014). In *H. intermedius*, pigmentation intensifies progressively throughout development, concentrating mainly along the dorsal and lateral regions of the body. This pattern has also been reported in other species of the same genus, such as *H*. aff. *malabaricus* and *H. lacerdae*, in which pigmentation intensifies especially in the postflexion stage (Nakatani et al. 2001; Gomes et al. 2010). As these species build nests close to riparian vegetation, larvae develop a disruptive pigmentation pattern that provides perfect mimicry with the substrate of these environments (Machado‐Allison 2020). This coloration strategy allows them to remain unnoticed, enhancing crypsis and facilitating prey capture (Gomes et al. 2010).

Because pigmentation patterns are widely used for larval identification, other morphological traits also become important for distinguishing taxa during early development. In Characiformes, larvae of the genus *Hoplias* can be recognized by a combination of features, including a fusiform body, relatively large head and eyes, terminal mouth, and a long intestine, with the anal opening positioned posterior to the vertical passing through the midbody (Bialetzki et al. 2008; Garcia et al. 2016). However, species‐level identification within the genus is more complex because several characters, such as myomere counts and fin‐ray, overlap among congeners (Bialetzki et al. 2008). Therefore, species identification generally relies on the combined interpretation of multiple morphological traits rather than on a single diagnostic character.

In addition to these morphological traits, meristic data are also fundamental for the early identification and differentiation. of fish larvae. *Hoplias intermedius* exhibited 36 to 44 myomeres, compared with 37–45 in *H*. aff. *malabaricus* (Bialetzki et al. 2008). Regarding fin‐rays counts, *H. intermedius* exhibited the following ranges: pectoral (9–10), pelvic (7–9), dorsal (12–14), anal (10–13), and caudal (18–20). In *H*. aff. *malabaricus*, the reported values were: pectoral (12), pelvic (7–9), dorsal (13–15), and anal (10–12) (Bialetzki et al. 2008). Although these meristic traits provide useful diagnostic information, the overlap in myomeres, fin‐ray counts and other developmental features among congeners indicates that these characters alone may not be sufficient for species identification.

**Figure 7:**
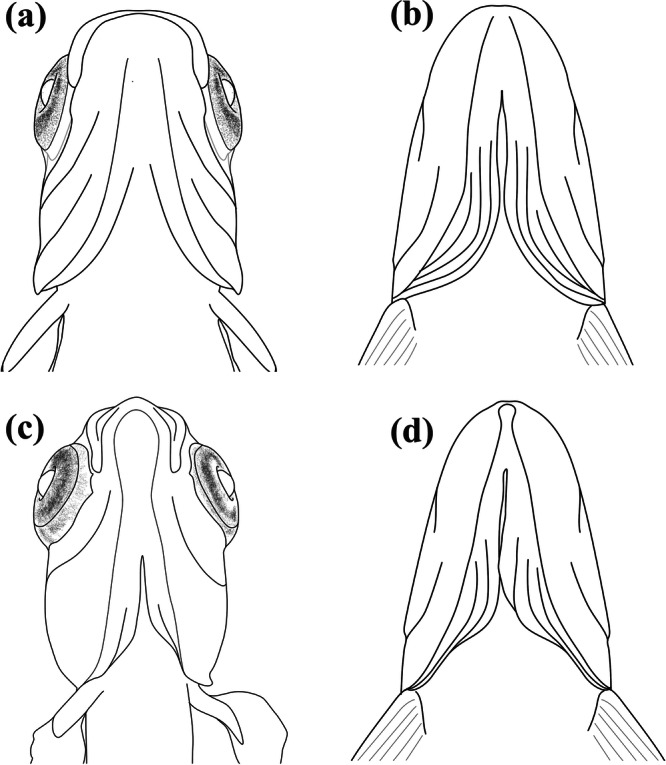
Lower jaw morphology of *Hoplias* larvae and adults. a Larva of *Hoplias intermedius*, showing the lower margins of the dentary bones converging toward the mandibular symphysis; b adult *H. intermedius*, with the same convergent pattern; and c larva of *H*. gr. *malabaricus*, showing the lower margins of the dentary diverging laterally at the anterior tip, d adult *H*. gr. *malabaricus* adult, exhibiting the same pattern. Illustrations in b and d were adapted from Britski et al. (1988).

However, Britski et al. (1988) noted that *H*. cf. *lacerdae* (= *H. intermedius*) exhibits the lower margins of the dentary bones converging (Figure 7b), sometimes subtly, toward the mandibular symphysis, whereas in *H. malabaricus* (= *H*. gr. *malabaricus*) this margin diverges laterally at the anterior tip (Figure 7d). This morphological feature (Figure 7) begins to be discernible at 8.1 mm SL during the preflexion stage, but becomes markedly evident from 9.56 mm SL onwards, at the flexion stage (Figure 7a), whereas in *H*. gr. *malabaricus* this character differentiates during the flexion stage at 9.40 mm SL (Figure 7c) making it a reliable character for distinguishing these taxa.

Ontogenetic analysis revealed breakpoints in morphometric traits such as body height, head dimensions, and pre‐fin distances, indicating shifts in growth priorities during early development. These discontinuities are commonly interpreted as markers of critical development events, including the onset of exogenous feeding or changes in locomotor performance, during which functions such as feeding, swimming, and respiration undergo reorganization (Osse 1989; Osse and Van Den Boogaart 1999; Osse et al. 1997). In *H*. *intermedius*, eye diameter and head depth exhibited accelerated growth, whereas snout and maxillary growth slowed. This contrast suggests a reallocation of developmental investment from prey‐capture structures toward features associated with enhanced visual capacity and cranial support (Webb and Weihs 1986; Osse 1989). Such patterns are consistent with broader ontogenetic trends observed in teleosts, in which positive allometry groeh of the head and tail is typically followed by near‐isometric growth that improves functional efficiency (Osse and Van Den Boogaart 1999; Santos et al. 2020; Oliveira et al. 2024; Silva et al. 2024). Thus, the breakpoints observed in *H*. *intermedius* not only reflect morphological reorganizations but also represent adaptive strategies aligned with the ecological demands of larval development.

Finally, our findings indicate that *H. intermedius* conforms to the expected developmental pattern of a species exhibiting parental care, large larvae, and slow development, traits that likely enhance reproductive success and survival. Morphometric changes, particularly during the flexion and postflexion stages and juvenile, corresponded to major behavioral and physiological shifts and refleting the altricial development. Characteristics such as a fusiform and heavily pigmented body, large eyes, and a long intestine are important for distinguishing this species from other Characiformes (Nakatani et al. 2001). These findings contribute not only to species identification and to the understanting of early development in Neotropical fishes, but also provide a foundation for conservation strategies and aquaculture management.

## 5 Conclusion

This study provided a detailed description of the early development of *Hoplias intermedius*, from the embryonic to the juvenile period, revealing morphological, meristic, and morphometric characteristics. Although meristic and morphometric traits may overlap with those of closely related species, specific morphological features, such as mouth position and lower jaw morphology, facilitate its distinction from congeners, particularly *H*. gr. *malabaricus*, with which it co‐occurs in the São Francisco River basin. The results demonstrated that *H*. *intermedius* exhibits an altricial developmental pattern, characterized by large, slow‐growing larvae, which is typical of species exhibiting parental care and high survival rates. Morphometric analyzes revealed ontogenetic changes marked by breakpoints associated with critical developmental events, such as the onset of exogenous feeding and increased swimming capacity, reflecting functional reorganizations and ecological adaptations throughout ontogeny. Overall, these characteristics contribute to the identification of the species in ichthyoplankton samples and improve the understanding of early development in Neotropical fish, providing valuable insights for conservation strategies and aquaculture management.

## Author Contributions

**Mateus Babichi Veiga de Souza:** conceptualization, investigation, writing – original draft, methodology, formal analysis, writing – review and editing, data curation, software. **Renan Souza Volpato:** conceptualization, methodology, investigation, writing – original draft. **Andréa Bialetzki:** conceptualization, methodology, data curation, investigation, formal analysis, supervision, writing – original draft, writing – review and editing.

## Ethics Statement

The collections and analyzes were conducted prior to the enforcement of the Brazilian guidelines for the care and use of animals in teaching or scientific research (Diretriz Brasileira para o Cuidado e a Utilização de Animais em Atividades de Ensino ou de Pesquisa Científica – DBCA), established by Resolution No. 55 of October 5, 2022, from the Conselho Nacional de Controle de Experimentação Animal (CONCEA). All procedures followed institutional standards and good practices to ensure animal welfare throughout the study. The DBCA and RN 55‐CONCEA are available at: https://www.in.gov.br/en/web/dou/-/resolucao-n-55-de-5-de-outubro-de-2022-434869177.

## Conflicts of Interest

The authors declare no conflict of interest.

## Data Availability

The data supporting the findings of this study are available from the corresponding author upon reasonable request.
